# Long‐term regeneration of a tropical plant community after sand mining

**DOI:** 10.1002/ece3.4111

**Published:** 2018-05-04

**Authors:** Mário L. Garbin, Flora Misaki, Poliana F. Ferreira, Karlo G. Guidoni‐Martins, Rayara B. Soares, Pierre Mariotte, Jerônimo B. B. Sansevero, Patryck Gouvea Rocha, Ary G. Silva

**Affiliations:** ^1^ Programa de Pós‐Graduação em Ecologia de Ecossistemas Universidade Vila Velha Vila Velha Brazil; ^2^ Programa de Pós‐Graduação em Ecologia e Evolução Universidade Federal de Goiás Goiânia Brazil; ^3^ School of Architecture, Civil and Environmental Engineering (ENAC) Laboratory of Ecological Systems (ECOS) Ecole Polytechnique Fédérale de Lausanne EPFL Lausanne Switzerland; ^4^ Snow and Landscape Research (WSL) Swiss Federal Institute for Forest Lausanne Switzerland; ^5^ Instituto de Florestas (IF) Departamento de Ciências Ambientais (DCA) Universidade Federal Rural do Rio de Janeiro (UFRRJ) Rio de Janeiro Brazil

**Keywords:** community assembly, disturbance, facilitation, natural regeneration, restinga, sand mining

## Abstract

Sandy coastal plant communities in tropical regions have been historically under strong anthropic pressure. In Brazil, these systems shelter communities with highly plastic plant species. However, the potential of these systems to regenerate without human assistance after disturbances has hardly been examined. We determined the natural regeneration of a coastal sandy plain vegetation (restinga) in Brazil, 16 years after the end of sand removal. We inventoried 38 plots: 20 within a sand‐mined site and 18 in an adjacent undisturbed site. We expected lower diversity values in the sand‐mined site compared to the undisturbed site, but similar species composition between the two sites due to the spatial proximity of the two sites and the high plasticity of restinga species. Species were ranked using abundance and importance value index in both sites, and comparisons were performed using Rényi entropy profiles, rarefaction curves, principal component analysis, and redundancy analysis. Species composition and dominant species differed markedly between the two sites. Bromeliads and *Clusia hilariana*, well‐known nurse plants, dominated the undisturbed site but were almost absent in the regenerating site. Species richness did not differ between both sites, but diversity was higher in the undisturbed site. Within‐site composition differences in the mined area were associated with field characteristics. Interestingly, species classified as subordinate or rare in the undisturbed site became dominants in the regenerating site. These newer dominants in the sand‐mined site are not those known as nurse plants in other restingas, thus yielding strong implications for restoration.

## 1 INTRODUCTION

The Atlantic forest is one of the main hotspots of biodiversity in the world with the highest conservation priorities (Morellato & Haddad, 2000; Myers, Mittermeier, Mittermeier, da Fonseca, & Kent, 2000). While the Atlantic forest was covering most of eastern Brazil prior to European colonization, it has now been reduced to only 7%–8% of its original area (Fundação SOS Mata Atlântica, INPE, 2011) and to 11.4%–16% if secondary forests and small fragments are considered (Ribeiro, Metzger, Martensen, Ponzoni, & Hirota, 2009). The coastal vegetation of this biome has a long history of human occupation starting eight thousand years ago (Calippo, 2008). Today, more than 26% of the Brazilian population, about 50.7 million people, lives in the coast (IBGE, 2011). Urbanization, seaside resorts, real estate speculation, agricultural activities, and sand mining are the main threats to these coastal ecosystems. The coastal rain forest (sensu Oliveira‐Filho & Fontes, 2000) is composed out of different plant communities that can be classified into the core formation, the mesic rain forest, and the peripheral communities, such as high altitude campos, swamp forests, and restingas (Scarano, 2002). Restingas are the denomination of the Quaternary sandy coastal plains in Brazil, encompassing the geomorphological features and the vegetation that covers these plains (Araujo & Pereira, 2004; Zamith & Scarano, 2006). The geomorphological diversity (caused by changes in sea level) of these areas leads to a wide variety of vegetation types ranging from forests, scrub, and herbaceous plant communities, usually separated in a sharp zonation pattern (Araujo & Pereira, 2004; Pimentel et al., 2007). Restingas possess a low number of endemic species compared to the core formation but are the reservoir of important ecological, evolutionary, and adaptive processes (Scarano, 2009). For instance, due to high plasticity and adaptations to water and nutrient stress under the forest canopy, rare epiphytes and hemiepiphytes species of the core formation may become terrestrial dominants in the peripheral restingas, where conditions are more extremes in terms of drought, salinity, temperature, and fertility (Scarano, 2002). Such plasticity and adaptations are also expected to positively affect the natural regeneration of degraded restingas, but this has not been tested yet.

Some of the stress‐adapted species in the restingas are considered as nurse plants, such as the dominant tree *Clusia hilariana* Schltdl. with its role in ameliorating stressful environmental conditions (Dias & Scarano, 2007). This nurse tree attains dominance by its higher capacity to tolerate water deficits (Rosado & de Mattos, 2010) and by its higher production of diaspores per flower than subordinate species (Garbin et al., 2016). Bromeliads are also abundant and provide safe germination sites for other plant species (Scarano, 2006; Scarano et al., 2004). Positive interactions are common in restinga communities and explain, for example, the association between understory trees and isolated adult trees (Castanho, Oliveira, & Prado, 2012). Nevertheless, such positive effects can become neutral or even negative under extreme environmental conditions (Castanho, Oliveira, & Prado, 2015), being strongly dependent on life form (e.g., herbs, shrubs, trees, and climbers). Scarano et al. (2004) proposed a general framework for the community dynamics of the open restinga vegetation, dominated by *C. hilariana*. In their model, the sandy substrate is initially colonized by *Aechmea nudicaulis* (a bromeliad) or by *Allagoptera arenaria* (a geophytic palm). Such pioneer species would provide better conditions for the establishment of *C. hilariana*, which would then facilitate the establishment of other plant species underneath its canopy. It is unclear, however, whether dominant nurse plants, such as *Clusia* and bromeliads, can nucleate the regeneration of disturbed sites of the restingas. Scarano (2009) hypothesized that locally rare or subordinate species might become more abundant after disturbances in the peripheral systems of the Atlantic rain forest and increase in abundance after disturbances, such as fire (Cirne & Scarano, 2001), probably due to strong clonal growth.

Restingas are located outside the main core formation of the Atlantic rain forest, and despite high threats in these areas, they are often neglected in conservation policies (Scarano, 2009). Therefore, these ecosystems are understudied, and we know little about their capacity to regenerate after disturbances. Previous studies found no evidence of recovery to wood extraction in terms of tree species richness, even 10 years after the perturbation (Scarano, Rios, & Esteves, 1998). The removal of locally rare trees led to the death of bromeliads, which were germination sites for the trees themselves (Scarano, 2006). Such findings highlight the fragility and the low resilience of these peripheral ecosystems and call for better understanding of the mechanisms driving community assembly in order to provide efficient restoration and conservation strategies. Active restoration (e.g., native tree and shrub plantation) has been successful in the restingas, upon condition that exotic grass is removed (Zamith & Scarano, 2006), and in swamp forests despite high interspecific variation in the responses to plantation (Zamith & Scarano, 2010). Unfortunately, as mentioned above, restoration of restinga plant communities is not a priority and is also relatively expensive, especially in highly disturbed areas. The question remains of whether there is a role for passive restoration (e.g., natural regeneration, see Holl & Aide, 2011) in disturbed restingas of the Atlantic forest.

Vegetation surveys in naturally recovering areas are an important tool for identifying framework species, that is, native species that could be planted to accelerate natural regeneration and encourage biodiversity recovery on degraded sites (Blakesley et al., 2002; Dias et al., 2014; Elliot et al., 2002; Elliott, 2003). In this regard, species richness represents the most frequent indicator in studies that measure restoration success (see Wortley, Hero, & Howes, 2013). In a recent global meta‐analysis, species richness was considered as the main indicator to quantify how far restoration projects are from reference ecosystems (see Crouzeilles et al., 2016) and it is associated with ecosystem functioning (Aerts & Honnay, 2011; Bu, Zang, & Ding, 2014; Liang et al., 2016).

In this study, we aimed at determining long‐term vegetation recovery of a sand‐mined site in restinga vegetation in Brazil, 16 years after the end of the disturbance. Here, we consider plant community composition and species diversity as two indicators of the recovery of ecosystem functions. We determined the recovery of plant community composition and species diversity using vegetation surveys carried out in undisturbed and sand‐mined sites. Vegetation recovery is generally slow in tropical coastal plant communities, and we expected that the diversity and abundance values were still low in the mined site, even 16 years after the end of the perturbation. However, due to clonal regrowth and spatial proximity between the two sites, we expected similar floristic composition (in terms of species and life forms) in mined and undisturbed sites. Our goals were to assess the success of passive restoration by identifying which species recolonize the sand‐mined site and by estimating the degree of plant community recovery after disturbance. We also discuss the implications of our findings for restoration and conservation strategies in the peripheral ecosystems of the Atlantic forest.

## 2 METHODS

### 2.1 Study site and sampling

The study was conducted in the Paulo César Vinha State Park (1,574.85 ha), in the municipality of Guarapari (20°33′S and 40°26′W), located in a sandy coastal plain plant community in southeast Brazil. The park was created in 1990 under the designation of Setiba State Park, and it was renamed as Paulo César Vinha State Park in 1994 (http://www.meioambiente.es.gov.br/), in honor of the environmentalist Paulo César Vinha, killed for denouncing the sand‐mining activities within the park. Sand removal (probably by backhoe) ended in 1994. A large sand‐mined area covering about 1.2 ha (maximum 3.25 m depth) and with exposed clayish substrate from Tertiary sediments (Teixeira, Gillison, & Silva, 2014) was chosen as a representative site for the study.

We used vegetation data from Ferreira and Silva (2014). Briefly, sampling took place between April and July 2010. Thirty‐eight 10 m × 10 m plots were inventoried: 20 within the sand‐mined site and 18 in an adjacent undisturbed site, which was used as reference ecosystem (sensu White & Walker, 1997), totaling 2,000 m^2^ and 1,800 m^2^ of the sampled area for each site, respectively. Plots in the sand‐mined site were distributed from the center of the mined area corresponding to more intense mining disturbance to the edges corresponding to lower mining disturbance. The minimum distance between the two sites was of 20 m. All plant species, regardless of the life form (trees, shrubs, and herbs), with a minimum of 1.5 cm of diameter at the soil level were sampled in each plot. We used three variables to describe each plot within the sand‐mined site: the slope (inclination of the terrain, in degrees), distance from the plot to the center of the mined area (i.e., “distance”), and depth (the vertical distance between the ground level at the edge of the mined area and the plot).

### 2.2 Data analysis

All analyses were carried out in the R environment (R Development Core Team, 2015) using the vegan package (Oksanen et al., 2015). We began our analytical procedures by running a multivariate sample sufficiency test (Anderson & Santana‐Garcon, 2015) using the Bray–Curtis distance in log‐transformed abundances for the two sets of plots (sand‐mined and undisturbed). Then, we plotted rank–abundance curves (Magurran, 2004; Whittaker, 1965) for sand‐mined and undisturbed sites. This procedure allowed us to identify graphically different components of species diversity. The length of the tail indicated the number of species, while equitability is checked by curve inclination; the more the curve is inclined, the lower is its equitability (Magurran, 2004; Melo, 2008). Further, Rényi diversity profiles (Anand & Orlóci, 1996; Rényi, 1961; Tóthmérész, 1995) were built for sand‐mined and undisturbed sites. The most common diversity indices, such as Shannon and Simpson, are special cases of the Rényi generalized entropy:

$$H_{\alpha }=\frac{1}{1-\alpha }\ln\sum_{i=1}^{S}p_{i}^{\alpha }$$

where *H* is the value of Rényi entropy for a given α and *p*_*i*_ is the proportional representation of each component *S*. Increasing values of α will return different measures of diversity, each one giving more weight to the abundance of component species. When α = 0, *H* corresponds to the logarithm of the richness. When α gets closer to 1, *H* tends toward the Shannon diversity index, whereas when α = 2, *H* corresponds to the inverse of the Simpson index (1/*D*). High‐order entropy values are preferable because it is where *H* reaches stability (Anand & Orlóci, 1996; Duarte, Machado, Hartz, & Pillar, 2006). A community will be considered more diverse than another community when all its Rényi entropy values are higher than those of the other community (Tóthmérész, 1995). Rarefaction curves were used to test whether sites differ in species richness irrespective of differences in plant abundance (Gotelli & Colwell, 2001).

Moreover, species were ranked by their importance value index (IVI, Brower, Zar, & von Ende, 1998; Dias, de Mattos, Vieira, Azeredo, & Scarano, 2006) for sand‐mined and undisturbed sites in order to identify dominant, subordinate, and transient species in the sites (see Grime, 1998; Mariotte, 2014; Whittaker, 1965). This was made within each life form (trees/shrubs and herbs) and was based on the sum of three components: relative frequency, relative density, and relative abundance values of each species.

Principal component analysis (PCA) was used to identify the main trends in variation of species composition in both sites. Abundance data were Hellinger‐transformed prior to ordination analysis (Legendre & Gallagher, 2001). We used a permutation multivariate ANOVA (PERMANOVA; Anderson, 2001) to test the differences in plant composition between undisturbed and sand‐mined sites, coded as factors. This analysis was run on a Bray–Curtis distance of a log_2_‐transformed abundance matrix. Between‐site differences may be due to differences in location of the factors in the multivariate space of ordination (i.e., suggesting a local effect), or to the dispersion of the values in relation to the centroid within each factor (i.e., suggesting a high beta diversity within sites), or both (Anderson, Ellingsen, & McArdle, 2006; Warton, Wright, & Wang, 2012). To check this, we ran a permutation analysis of multivariate dispersion (PERMDISP; Anderson et al., 2006) on the same log_2_‐transformed abundance matrix using Bray–Curtis distance. Finally, a redundancy analysis (RDA; Legendre & Legendre, 2012) was run between species abundances × plot matrix (using only the 20 plots within the sand‐mined area) constrained by three variables describing terrain features of the sand‐mined area: slope (in degrees), depth (in meters), and distance from the center of the mined area (in meters). These field features were standardized (scaled to zero mean and unit variance) prior to analysis (McGarigal, Cushman, & Stafford, 2000), and absolute abundances of species data were log‐transformed prior to analysis (Legendre & Legendre, 2012). We used the vegan's *ordistep* function (Borcard, Gillet, & Legendre, 2011) to select the most related field features to the variation in the community matrix. Given the fact that there is only one site in each disturbance level, a case of pseudoreplication (Hurlbert,^1984^), the scope of our comparisons is limited to the local conditions tested. Thus, we use general knowledge to discuss our findings in comparison with other locations where the conditions are typical (see Webster, 2007), and we emphasize the importance to use our main conclusions as new hypotheses to be tested (see Davies & Gray, 2015) in other tropical coastal vegetations.

## 3 RESULTS

**Figure 1:**
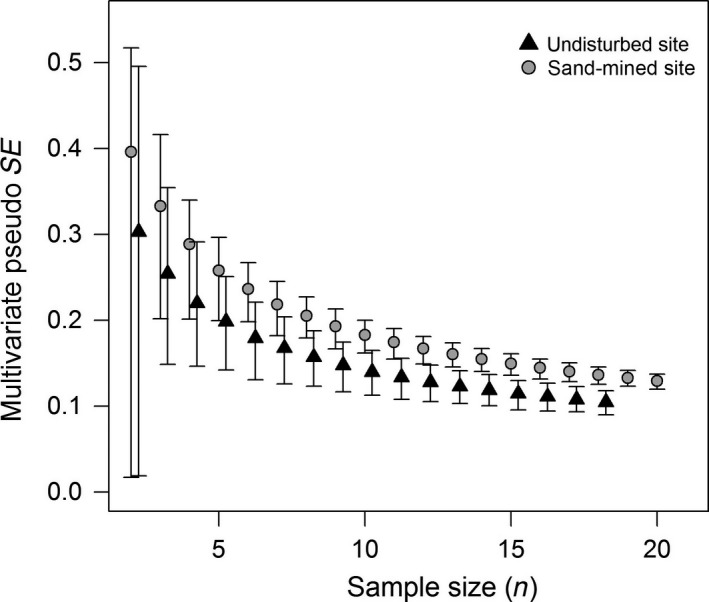
Result of the multivariate sample sufficiency procedure based on Bray–Curtis distance. Sample size sufficiency is given by both the stability in the curve (mean multivariate pseudo standard error) and reduction in the envelope sizes (percentiles, 2.5 and 97.5, after bootstrapping)

**Figure 2:**
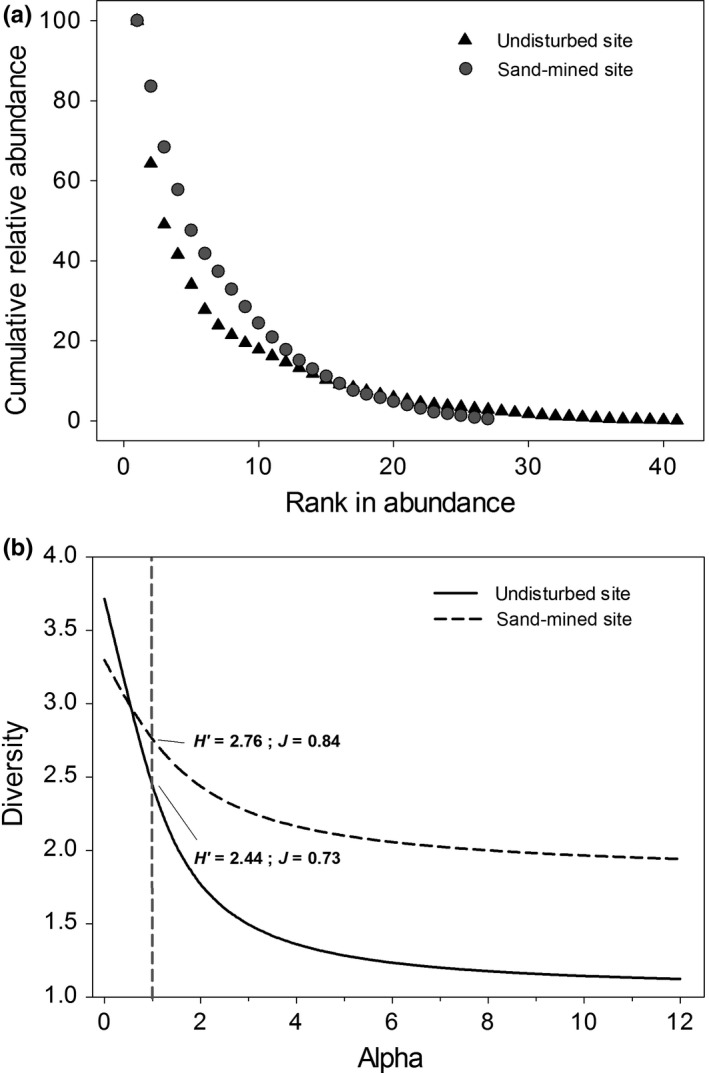
Abundance and diversity patterns of the undisturbed and sand‐mined sites in the coastal sandy plant community. (a) Changes in relative plant abundance in the undisturbed and sand‐mined sites. The *y*‐axis shows the cumulative relative abundance (species ranked by relative cover in the ascending order and cumulated). The *x*‐axis shows the species rank in the descending order, from the most to the less abundant plant species. The steepest slope of the undisturbed site dominance profile highlights higher plant dominance and thus lower diversity than the sand‐mined site. Calculations are independent for each site. (b) Diversity profile using the Rényi series. Dashed gray line indicates α = 1, which corresponds to the Shannon's diversity (*H*′). The undisturbed site was species rich, but its lower equitability (*J*) lowered the diversity outputs for α‐values higher than 0.5

**Figure 3:**
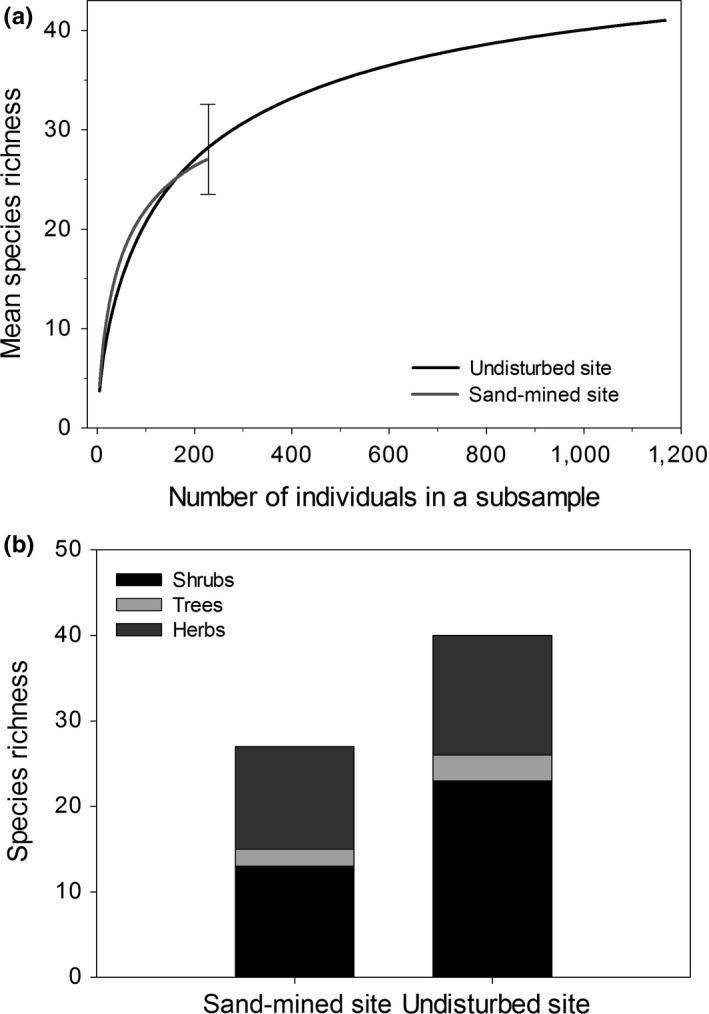
Rarefaction curves and species richness of the life forms in undisturbed and sand‐mined sites in the coastal sandy plant community. (a) Vertical line shows confidence interval (±2 *SE*) for the number of individuals in a sample of the undisturbed site. The species richness did not differ between undisturbed and sand‐mined sites when the number of individuals sampled in these sites is taken into account. (b) Differences in species richness relative to plant life forms. The richness of shrub species is higher in the undisturbed area than in the sand‐mined area

The multivariate sample sufficiency test showed that the number of plots in each site was sufficient to compare both sites, given the stability of the curve and the reduction in the envelope size (Figure 1). Sand‐mined and undisturbed sites differed in the shape of their rank–abundance curves (Figure 2a). The curve of sand‐mined site was less inclined than that of the undisturbed site, thus reflecting a higher evenness. The total number of species was higher in the undisturbed site (41 species) by comparison with the sand‐mined site (27 species). However, diversity was higher in the sand‐mined site due to the lower equitability of the undisturbed site (Figure 2b). Nonetheless, the rarefaction curves showed that there was no difference in species richness between the sites when controlling for density effects (Figure 3a), despite higher shrub species richness in the undisturbed site (Figure 3b).

**Figure 4:**
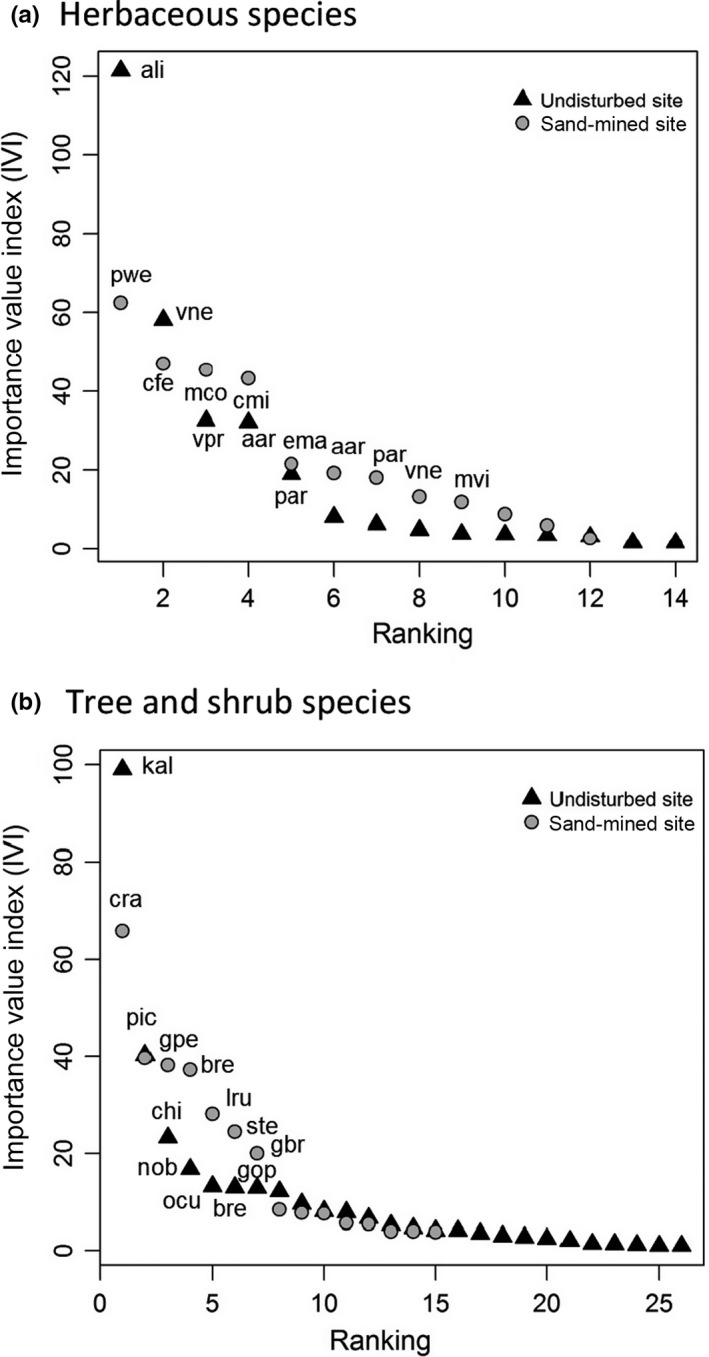
Importance value index (IVI) curves for (a) herbaceous/climber and (b) tree/shrub species in the undisturbed and sand‐mined sites. The disturbance in the sand‐mined site seems to affect the community structure reducing the relative frequency, relative abundance, and relative density of species, irrespectively of life forms. Codes for herbaceous species are as follows: ali, *Aechmea lingulata* (L.) Baker; aar, *Allagoptera arenaria* (Gomes) Kuntze; cfe, *Cereus fernambucensis* Lem.; cim, *Comanthera imbricata* (Körn.) L.R. Parra and Giul.; Ema, *Evolvulus_maximiliani* Mart.; mco, *Microstachys corniculata* (Vahl) Griseb; mvi, *Melocactus_violaceus* Pfeiff; par, *Pilosocereus arrabidae* (Lem.) Byles and Rowley; pwe, *Paullinia weinmanniifolia*; vne, *Vriesea neoglutinosa* Mez; vpr, *Vriesea procera* (Mart. ex Schult. and Schult.f.) Wittm. Codes for shrubs/tree species are as follows: bre, *Baccharis reticularia* DC.; chi, *Clusia hilariana* Schltdl.; cra, *Chamaecrista ramosa* (Vogel) H.S.Irwin and Barneby; gop, *Guapira opposita* (Vell.) Reitz; gpe, *Guapira pernambucensis* (Casar.) Lundell; kal, *Kielmeyera albopunctata* Saddi; lru, *Lepidaploa rufogrisea* (A.St.‐Hil.) H.Rob.; nob, *Neomitranthes obtuse* Sobral and Zambom; ono, *Ocotea notata* (Nees and Mart.) Mez; ocu, *Ouratea cuspidata* (A.St.‐Hil.) Engl.; pic, *Protium icicariba* (DC.) Marchand.; ste, *Schinus terebinthifolius* Raddi

Sand‐mined and undisturbed sites differed in the shape of their rank IVI curves and in the identity of dominant and subordinate species for both herb (Figure 4a) and tree/shrub species (Figure 4b). In the undisturbed site, herbaceous dominant species (*n* = 2) were the bromeliads, *Aechmea lingulata* and *Vriesea neoglutinosa*, and herbaceous subordinate species (*n* = 3) were *Vriesea procera* (bromeliad), *Allagoptera arenaria* (geophyte palm), and *Pilosocereus arrabidae* (cactus). In the sand‐mined site, herbaceous dominants (*n* = 4) were the climbing plant *Paullinia weinmanniifolia* (scandent vine), the cactus *Cereus fernambucensis*, the spurge *Microstachys corniculata*, and the pipewort *Comanthera imbricata* (Figure 4a). In the undisturbed site, dominant tree/shrub species (*n* = 2; Figure 4b) were the shrub *Kielmeyera albopunctata* and the tree *Protium icicariba*, while the subordinates (*n* = 6) were the tree species *Clusia hilariana*,* Neomitranthes obtusa*, and *Ouratea cuspidata* and the shrubs *Baccharis reticularia*,* Guapira pernambucensis*, and *Guapira opposita*. In the sand‐mined site, the dominant shrub species (*n* = 1) was *Chamaecrista ramosa* and the subordinate tree/shrub species (*n* = 6) were *Ocotea notata*,* Guapira pernambucensis*,* Baccharis reticularia*,* Lepidaploa rufogrisea*,* Schinus terebinthifolius*, and *Garcinia brasiliensis*. See Appendix for the list of species, abundances, frequencies, and IVI values in both sites.

**Figure 5:**
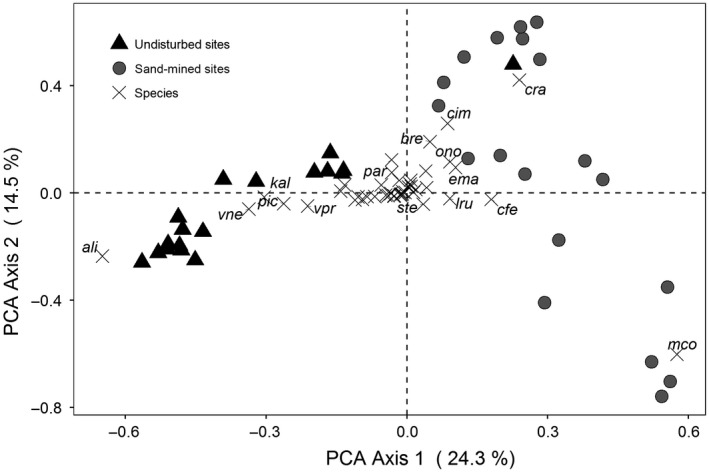
Principal component analysis of plots in sand‐mined (gray circles) and undisturbed (black triangles) described by the abundances of 45 herbaceous, shrub, tree and climber species. Axis 1 shows strong discrimination between undisturbed and sand‐mined sites, while axis 2 shows differences within the sand‐mined site with a contrast between plots dominated by *C. imbricata* and plots dominated by *M. corniculata. C. ramosa* dominated a single plot within the undisturbed site. Codes for species are the same as in Figure 4

**Figure 6:**
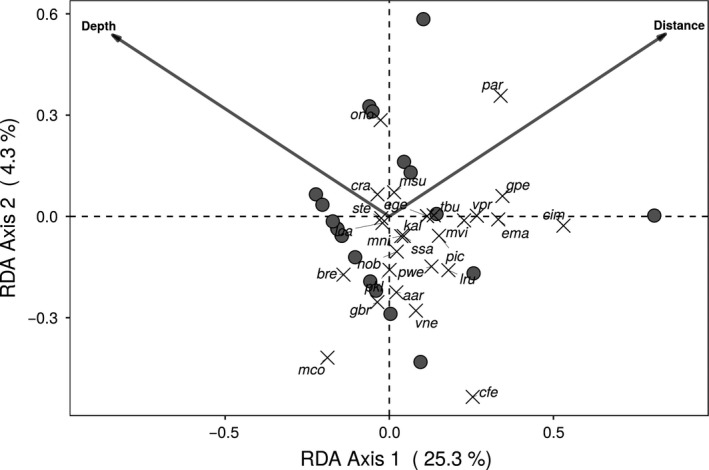
Redundancy analysis of the sand‐mined plots described by the species composition matrix in relation to selected field characteristics (depth and distance to the center of the mined area). Sand removal in some plots created deeper holes and exposed the sediments from the Tertiary layer, which both favored water accumulation and increased the abundance of *M. corniculata*. At the opposite, *C*. *imbricata* dominated the slopes within the sand‐mined site and was more abundant in plot with long distance between the edges and the center of the mined area. Codes for species are the same as in Figure 4, except for the following: msu, *Manilkara subsericea* (Mart.) Dubard; lca, *Lantana camara* L.; gbr, *Garcinia brasiliensis* Mart.; pkl, *Phyllanthus klotzschianus* Müll.Arg.; ssa, *Serjania salzmanniana* Schltdl.; mni, *Melanopsidium nigrum* Colla; ege, *Evolvulus genistoides* Ooststr.; tbu, *Tocoyena bullata* (Vell.) Mart

The differences in composition and abundance between the two sites were significant (PERMANOVA, *p *<* *.001) and are shown in the first two axes (39%) of the ordination graph (Figure 5). Moreover, composition differences between the sites can also be attributed to the within‐site dispersion in beta diversity (PERMDISP, *p *<* *.001). PCA axis 1 showed that different species dominated the sand‐mined and the undisturbed sites. However, PCA axis 2 showed that sand removal did not produce the same effects on all plots within the sand‐mined site with plots dominated by *M. corniculata* and other by *C. imbricata*, although both species were initially rare or subordinate species according to the reference site data. Field attributes (“depth” and “distance”) were significantly associated with the compositional patterns within the sand‐mined site, as shown by the RDA (*p *=* *.001; Figure 6), and show that the abundance of *M. corniculata* was higher in the deeper portions of the mined area (i.e., where sand has been more deeply mined). Conversely, *C. imbricata* was more abundant with increasing distance from the center of the sand‐mined area where mining disturbance was less important.

## 4 DISCUSSION

Previously exploited sand‐mined areas differed from undisturbed site in terms of plant species composition patterns, even 16 years after the end of the disturbance. This confirms previous findings highlighting the slow vegetation recovery following sand mining (Partridge, 1992). While species richness recovered, dominance was higher in the sand‐mined site and the identity of dominant and subordinate species greatly differed from the reference site. The regeneration of the sand‐mined site was principally driven by the increased abundance of the original subordinate or transient species (i.e., those of the preserved site), while well‐known nurse plants were absent or present in very low abundances. Such findings point to the importance of taking plant community floristic composition into account, indicating that passive restoration alone may not suffice and that human intervention may become necessary.

Resprouting often drives plant community composition after disturbances (Cirne & Scarano, 2001; Simões & Marques, 2007). Sand removal may have affected the potential for natural regeneration through resprouting. Moreover, local conditions such as depth (a proxy for bottom watering conditions; the deeper is the mining, the less well drained is the soil) and distance to the center of the mined area explained the differences in plant community composition, within the recovering sand‐mined site. Only the initially subordinate or rare species were able to colonize the center of the mined area with exposed Tertiary sediments. These findings suggest that differences in soil conditions between Quaternary (i.e., undisturbed site) and Tertiary sediments (i.e., sand‐mined site), and water availability are crucial for effective plant colonization of mined areas. Future studies should therefore determine the performance of the dominant and subordinate species in different types of soil (Tertiary vs. Quaternary) and moisture status (drained vs. water accumulation). This could help understand why dominant species of the undisturbed site did not successfully establish in the mined area and to identify framework species, which can accelerate plant recolonization of the mined site, especially in the slopes of the mined area.

Interestingly, the species that are becoming dominants in the sand‐mined site are not those for what we have knowledge about their facilitative effects (i.e., nurse species) and this has implications for the conservation and restoration of these coastal sandy plains. Plant facilitation can have a crucial role in restoration ecology (Brooker et al., 2007) by positively affecting the emergence and survival of plant species (Gómez‐Aparicio, 2009) or increasing diversity at intermediary‐to‐high environmental severity conditions (Castanho et al., 2015; Michalet et al., 2006). However, facilitation will be irrelevant if the plant species known to have such potential positive effect in undisturbed sites are absent from restoring sites. In arid Mediterranean ecosystems, facilitation may be less important than environmental heterogeneity and plant plasticity in affecting restoration (Valladares & Gianoli, 2007). Species that have positive effects on the abundances of other species in the restingas are the bromeliads (especially *Aechmea nudicaulis*), the tree *Clusia hilariana* (Dias & Scarano, 2007; Scarano, Ribeiro, de Moraes, & de Lima, 1997; Scarano et al., 2004), and the shrub *Erythroxylum subsessile* (Garbin, Carrijo, Sansevero, Sánchez‐Tapia, & Scarano, 2012; Garbin, Sánchez‐Tapia, Carrijo, Sansevero, & Scarano, 2014). In our study, facilitative species such as bromeliads and nurse shrubs became rare or subordinate species in the sand‐mined site, while the colonizing species were the subordinate shrubs and herb species *M. corniculata*,* C. imbricata* (Eriocaulaceae), and *C. ramosa* (Fabaceae). Here, this new combination of species in the mined area, led by anthropic activities, may be the result of an arrested succession caused by biotic or abiotic conditions (Boyes, Gunton, Griffiths, & Lawes, 2011; Brown & Lugo, 1994; Sarmiento, 1997) or a novel ecosystem (Hobbs, Higgs, & Hall, 2013; Hobbs, Higgs, & Harris, 2009; Hobbs et al., 2006), which generates different ecosystem processes, potentially driven by the initially subordinate or rare species. These formerly rare/subordinate species that are colonizing the sand‐mined area do not seem to produce the well‐documented nurse effects of the dominants in undisturbed restingas. In addition, the role of these newly dominant species on ecosystem functioning and in facilitating other species remains unknown in the restingas. Therefore, our findings raise the concern that important ecological processes, such as facilitation, may be lost in a scenario of human disturbed landscapes in coastal ecosystems. Nevertheless, the potential of the species colonizing the mined area to become nurse plants needs to be evaluated, especially because the dominant species in the mined area, *Chamaecrista ramosa*, is a Fabaceae, thus having a potential to be a nitrogen‐fixing species.

Community‐level patterns and processes are crucial to understand and manage natural systems (Simberloff, 2004). This is because management is, basically, a local activity. Restoration of the highly impacted coastal plant communities is an urgent demand in many parts of the world, and positive interactions can help to achieve this target (Lithgow et al., 2013). We show that the colonization of the disturbed site by well‐known nurse plants was not achieved after 16 years of disturbance, thus emphasizing the need for active restoration. The proper selection of framework species will be crucial in this regard (see Dias et al., 2014), and the role of these naturally recovering species and well‐known nurse plants as framework species will need to be tested along with the management of critical abiotic factors.

Our results raise the hypothesis that passive restoration of sandy coastal plains degraded by sand mining may not rely on the positive effects of typical nurse plants due to their absence or very low abundances in mined areas, even 16 years after disturbance. The long‐term results found here point to the need to test these findings across a larger range of sand‐mined sites. Rare or subordinate species in the undisturbed site became abundant in the mined area, and we suggest that these species should be screened in the future for their potential role as nurse plants. Moreover, diversity and richness alone were not good indicators of success because composition was quite different between regenerating and reference sites and well‐known nurse plants were absent from the regenerating site. Lastly, active restoration should be tested in sand‐mined sites by planting the nurse tree *Clusia hilariana* and/or by managing critical abiotic factors.

## CONFLICT OF INTEREST

None declared.

## AUTHOR CONTRIBUTIONS

MG and AS conceived and designed the research; PF, RB, PR, and AS performed the field survey and species' identifications; MG, KM, and FM analyzed the data; MG, FM, KG‐M, JS, and PM wrote and edited the manuscript.

## APPENDIX 1

### Phytosociological table for herbaceous/climber and tree/shrub species in the undisturbed and sand‐mined sites. IVI, importance value index

**Table:**

Site condition	Life form	Species	Absolute density	Relative density	Absolute frequency	Relative frequency	Absolute dominance	Relative dominance	IVI
Sand‐mined	Herbs	*Paullinia weinmanniifolia*	2	1.39	10.00	3.85	0.16	57.16	62.40
		*Cereus fernambucensis*	23	15.97	40.00	15.38	0.04	15.65	47.01
		*Microstachys corniculata*	34	23.61	45.00	17.31	0.01	4.59	45.51
		*Comanthera imbricata*	37	25.69	30.00	11.54	0.02	6.07	43.31
		*Evolvulus maximiliani*	13	9.03	30.00	11.54	0.00	0.98	21.55
		*Allagoptera arenaria*	7	4.86	25.00	9.62	0.01	4.72	19.19
		*Pilosocereus arrabidae*	9	6.25	20.00	7.69	0.01	4.14	18.08
		*Vriesea neoglutinosa*	6	4.17	20.00	7.69	0.00	1.42	13.27
		*Melocactus violaceus*	4	2.78	15.00	5.77	0.01	3.41	11.95
		*Serjania salzmanniana*	4	2.78	15.00	5.77	0.00	0.41	8.95
		*Vriesea procera*	4	2.78	5.00	1.92	0.00	1.38	6.09
		*Evolvulus genistoides*	1	0.69	5.00	1.92	0.00	0.07	2.69
	Trees/shrubs	*Chamaecrista ramosa*	24	29.63	68.75	24.44	0.01	11.80	65.87
		*Ocotea notata*	8	9.88	31.25	11.11	0.01	18.69	39.67
		*Guapira pernambucensis*	10	12.35	31.25	11.11	0.01	14.69	38.15
		*Baccharis reticularia*	10	12.35	25.00	8.89	0.01	16.03	37.26
		*Lepidaploa rufogrisea*	10	12.35	31.25	11.11	0.00	4.67	28.13
		*Schinus terebinthifolius*	1	1.23	6.25	2.22	0.01	20.95	24.41
		*Garcinia brasiliensis*	5	6.17	18.75	6.67	0.00	7.12	19.96
		*Protium icicariba*	2	2.47	12.50	4.44	0.00	1.47	8.39
		*Neomitranthes obtusa*	2	2.47	12.50	4.44	0.00	0.83	7.74
		*Tocoyena bullata*	2	2.47	12.50	4.44	0.00	0.68	7.59
		*Manilkara subsericea*	2	2.47	6.25	2.22	0.00	0.99	5.68
		*Phyllanthus klotzschianus*	2	2.47	6.25	2.22	0.00	0.77	5.47
		*Kielmeyera albopunctata*	1	1.23	6.25	2.22	0.00	0.50	3.95
		*Melanopsidium nigrum*	1	1.23	6.25	2.22	0.00	0.50	3.95
		*Lantana camara*	1	1.23	6.25	2.22	0.00	0.32	3.77
Undisturbed	Herbs	*Aechmea lingulata*	417	52.32	70.59	17.14	0.80	51.90	121.37
		*Vriesea neoglutinosa*	177	22.21	70.59	17.14	0.29	18.72	58.08
		*Vriesea procera*	88	11.04	41.18	10.00	0.18	11.58	32.62
		*Allagoptera arenaria*	46	5.77	88.24	21.43	0.08	4.94	32.14
		*Pilosocereus arrabidae*	19	2.38	35.29	8.57	0.13	8.09	19.04
		*Cereus fernambucensis*	10	1.25	23.53	5.71	0.02	1.22	8.19
		*Comanthera imbricata*	18	2.26	11.76	2.86	0.02	1.20	6.32
		*Evolvulus maximiliani*	4	0.50	17.65	4.29	0.00	0.14	4.92
		*Anthurium intermedium*	7	0.88	11.76	2.86	0.00	0.10	3.84
		*Melocactus violaceus*	3	0.38	5.88	1.43	0.03	1.84	3.64
		*Evolvulus genistoides*	4	0.50	11.76	2.86	0.00	0.08	3.44
		*Stachytarpheta cayennensis*	2	0.25	11.76	2.86	0.00	0.04	3.15
		*Hylocereus setaceus*	1	0.13	5.88	1.43	0.00	0.10	1.65
		*Serjania salzmanniana*	1	0.13	5.88	1.43	0.00	0.06	1.61
	Trees/shrubs	*Kielmeyera albopunctata*	88	23.85	88.89	11.43	0.78	63.76	99.04
		*Protium icicariba*	73	19.78	88.89	11.43	0.11	9.01	40.22
		*Clusia hilariana*	19	5.15	50.00	6.43	0.14	11.64	23.22
		*Neomitranthes obtusa*	28	7.59	50.00	6.43	0.03	2.72	16.74
		*Ouratea cuspidata*	23	6.23	33.33	4.29	0.03	2.65	13.17
		*Baccharis reticularia*	17	4.61	50.00	6.43	0.02	1.89	12.93
		*Guapira pernambucensis*	17	4.61	55.56	7.14	0.01	1.05	12.80
		*Guapira opposita*	17	4.61	50.00	6.43	0.01	1.07	12.11
		*Myrciaria floribunda*	13	3.52	38.89	5.00	0.01	1.04	9.56
		*Tocoyena bullata*	10	2.71	38.89	5.00	0.00	0.38	8.09
		*Coccoloba arborescens*	10	2.71	33.33	4.29	0.01	0.84	7.84
		*Ocotea notata*	9	2.44	27.78	3.57	0.01	0.64	6.65
		*Melanopsidium nigrum*	8	2.17	22.22	2.86	0.00	0.22	5.25
		*Erythroxylum subsessile*	5	1.36	22.22	2.86	0.00	0.41	4.62
		*Manilkara subsericea*	4	1.08	22.22	2.86	0.00	0.18	4.13
		*Stigmaphyllon paralias*	4	1.08	22.22	2.86	0.00	0.14	4.08
		*Chamaecrista ramosa*	4	1.08	16.67	2.14	0.00	0.27	3.50
		*Andira nitida*	4	1.08	11.11	1.43	0.00	0.40	2.91
		*Garcinia brasiliensis*	4	1.08	11.11	1.43	0.00	0.20	2.72
		*Agarista revoluta*	3	0.81	5.56	0.71	0.01	0.89	2.41
		*Lepidaploa rufogrisea*	2	0.54	11.11	1.43	0.00	0.06	2.03
		*Anacardium occidentale*	2	0.54	5.56	0.71	0.00	0.15	1.40
		*Couepia ovalifolia*	2	0.54	5.56	0.71	0.00	0.13	1.38
		*Ternstroemia brasiliensis*	1	0.27	5.56	0.71	0.00	0.19	1.17
		*Phyllanthus klotzschianus*	1	0.27	5.56	0.71	0.00	0.03	1.02
		*Myrsine umbellata*	1	0.27	5.56	0.71	0.00	0.03	1.02
